# GinorMass: A Tandem Mass Spectral Repository of 24 Million Spectra From One Molecule

**DOI:** 10.1002/ansa.70107

**Published:** 2026-09-25

**Authors:** Linh Hoang, Gary Siuzdak

**Affiliations:** ^1^ Scripps Center for Metabolomics and Mass Spectrometry, Department of Integrative Structural and Computational Biology, and Department of Chemistry Scripps Research La Jolla California USA

Mass spectrometry (MS) repositories and reference libraries have become indispensable resources for metabolomics, exposomics, natural products research, and analytical chemistry [1, 2, 3, 4, 5, 6]. Recent computational advances now enable searching across billions of tandem mass spectra and spectral relationships within public repositories, representing a remarkable achievement in data indexing and retrieval. These advances have substantially expanded our ability to discover, compare, and reuse mass spectrometry data. However, repository scale should not be conflated with molecular knowledge. Spectral count and authenticated molecular identities represent fundamentally different quantities. Published descriptions of resources such as GNPS, MassBank, and HMDB routinely use the numbers of spectra, entries, datasets, or files to communicate scale [3, 4, 5, 6]. These metrics appropriately describe data volume, acquisition activity, and computational capability. However, spectral abundance, now often measured in the billions [6], does not by itself reflect the number, diversity, or confidence of the underlying molecular identities. To make this distinction experimentally explicit, we created GinorMass, a publicly downloadable collection [https://masspec.scripps.edu/] of 23,971,300 MS/MS spectra acquired from a single authenticated molecular standard: arginine.

GinorMass clarifies what each dimension represents. Tandem mass spectra (MS/MS) are analytical observations, whereas authenticated molecular identities represent experimentally anchored molecular knowledge. Replicate measurements may characterise analytical variability and reproducibility, but they do not expand molecular coverage, which grows only when additional compounds are characterised and validated (Figure 1) [7, 8, 9, 10].

**FIGURE 1:**
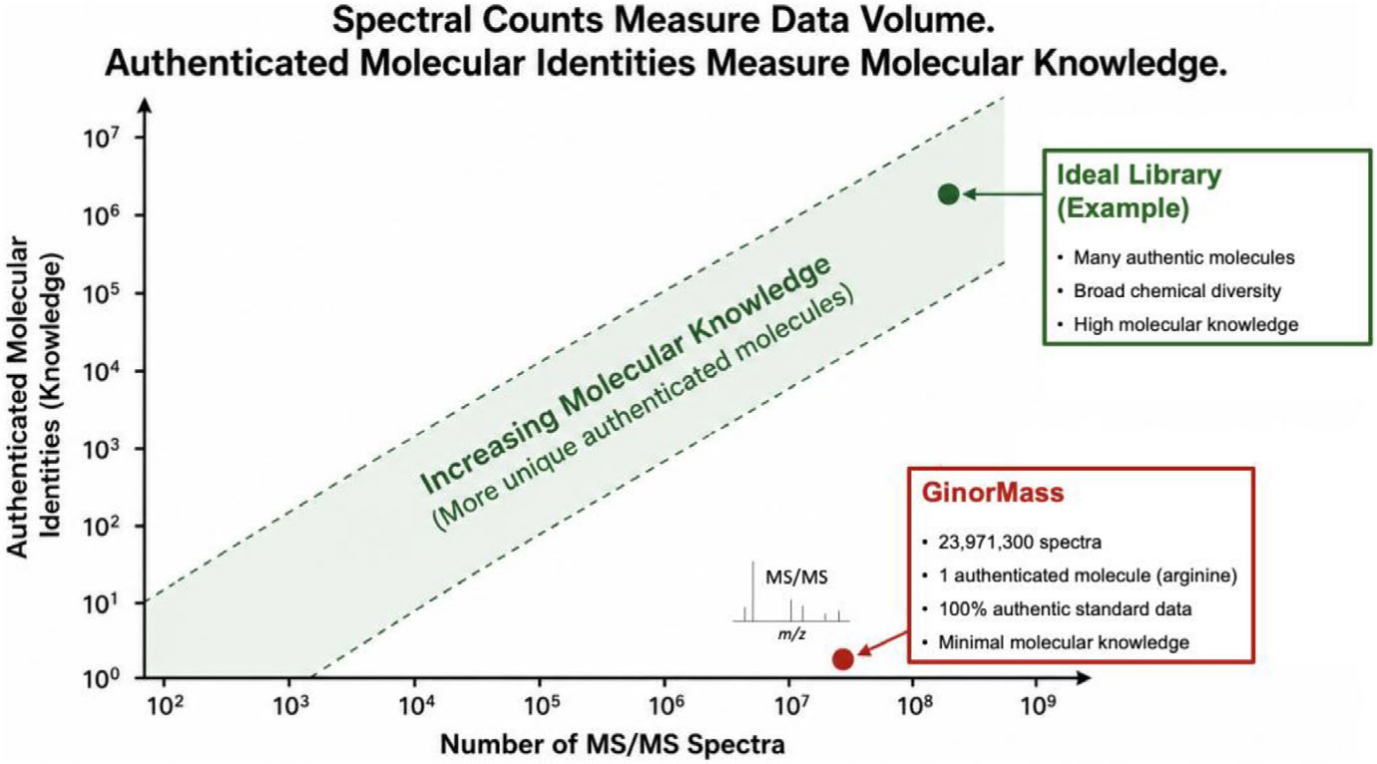
Spectral abundance and authenticated molecular diversity are distinct dimensions of mass spectral resource content. GinorMass (red) contains 23,971,300 experimentally acquired MS/MS spectra from one authenticated compound, arginine. A broadly diverse reference library (green) could contain a comparable number of spectra while representing many authenticated molecular identities. The “Ideal Library” is a conceptual example illustrating a resource that combines high spectral abundance with broad coverage of authenticated molecular identities; its position is illustrative and does not represent a specific repository or quantitatively defined ideal.

Arginine was selected as a stable, readily available, biologically relevant and well‐characterised reference standard. At 20 eV in positive‐ion mode, its spectrum contains five fragment ions, which fall within the typical range of fragments observed across the approximately 960,000 experimentally characterised molecules in METLIN [2]. Arginine therefore provides a reasonable example under typical acquisition conditions. More importantly, GinorMass was designed to distinguish accumulation of spectral observations from expansion of authenticated molecular diversity.

The practical value of additional spectra depends on purpose. Replicates collected across instruments, laboratories, collision energies, or sample environments can improve characterisation of variability, robustness and transferability. For identification of a known compound under a fixed condition, however, additional spectra eventually become increasingly redundant. GinorMass makes both points visible: repeated spectra may deepen analytical characterisation of arginine, but they do not broaden the chemistry contained in the resource. Spectral diversity within a molecule and molecular diversity across a resource should therefore be reported separately.

This distinction raises a broader question: what should the field report when describing spectral resources? Spectral count should remain, but it should be accompanied by complementary measures. At minimum, these should include: (i) the number of unique molecular identities at an explicitly stated structural level; (ii) the number authenticated using reference standards; (iii) chemical‐class coverage; (iv) precursor, adduct, polarity, collision‐energy and instrument coverage; (v) annotation confidence and provenance. Where extensive replication is present, a measure of spectral redundancy or effective spectral diversity would provide additional context.

These measures can be implemented using transparent, reproducible definitions. Unique identities may be counted at the connectivity level, for example using the first block of the InChIKey, while stereochemical identities can be reported separately. Chemical‐class diversity can be summarised using a documented chemical ontology and reported as both the number and distribution of represented classes. Biological relevance can be described through stated cross‐references to curated pathway, organism, endogenous‐metabolite, drug, natural‐product or exposure resources. Annotation confidence should follow an established framework, and provenance should link each spectrum to its molecular standard or source, acquisition conditions and processing history [7, 8, 9, 10, 11, 12, 13]. No single metric captures every purpose, but explicit reporting makes resources comparable without conflating data volume with chemical breadth.

This is not an argument against large repositories or extensive replication. The accumulation and sharing of spectra have transformed analytical science. Rather, GinorMass illustrates why the dimensions of resource value should be named accurately. Spectral count usefully measures data volume and computational scale, but it should be reported alongside authenticated molecular identities, chemical and analytical coverage, annotation confidence, provenance and spectral redundancy. GinorMass expresses the distinction in its simplest form: 23,971,300 experimentally acquired tandem mass spectra from one authenticated molecule.

## Author Contributions

**Linh Hoang**: data curation, investigation, visualization, validation and formal analysis. **Gary Siuzdak**: conceptualisation, investigation, funding acquisition, writing – original draft, methodology, validation, visualisation, writing – review and editing, project administration, formal analysis, supervision and resources.

## Conflicts of Interest

The authors declare no conflicts of interest.

## Data Availability

The data supporting this article, comprising 23,971,300 arginine MS/MS spectra, are publicly available through Scripps Research at https://masspec.scripps.edu/. Spectra were acquired from an authenticated arginine standard in positive‐ion mode using a Bruker Impact II QTOF. Data were collected at an acquisition time of 20–50 ms/spectrum and a collision energy of 20 eV. The arginine standard at 10 µg/mL was introduced into the instrument via syringe pump at a flow rate of 3 µL/min.
